# Developing a Tool to Assess the Capacity of Out-of-School Time Program Providers to Implement Policy, Systems, and Environmental Change

**DOI:** 10.5888/pcd13.160105

**Published:** 2016-08-11

**Authors:** Jennifer Leeman, Jonathan L. Blitstein, Joshua Goetz, Alexis Moore, Nell Tessman, Jean L. Wiecha

**Affiliations:** Author Affiliations: Jonathan L. Blitstein, Joshua Goetz, Jean L. Wiecha, RTI International, Inc, Research Triangle Park, North Carolina; Alexis Moore, Department of Health Behavior, Gilling’s School of Global Public Health, University of North Carolina, Chapel Hill, North Carolina; Nell Tessman, Alliance for a Healthier Generation, Portland, Oregon.

## Abstract

**Background:**

Little is known about public health practitioners’ capacity to change policies, systems, or environments (PSEs), in part due to the absence of measures. To address this need, we partnered with the Alliance for a Healthier Generation (Alliance) to develop and test a theory-derived measure of the capacity of out-of-school time program providers to improve students’ level of nutrition and physical activity through changes in PSEs.

**Community Context:**

The measure was developed and tested through an engaged partnership with staff working on the Alliance’s Healthy Out-of-School Time (HOST) Initiative. In total, approximately 2,000 sites nationwide are engaged in the HOST Initiative, which serves predominantly high-need children and youths.

**Methods:**

We partnered with the Alliance to conduct formative work that would help develop a survey that assessed attitudes/beliefs, social norms, external resources/supports, and self-efficacy. The survey was administered to providers of out-of-school time programs who were implementing the Alliance’s HOST Initiative.

**Outcome:**

Survey respondents were 185 out-of-school time program providers (53% response rate). Exploratory factor analysis yielded a 4-factor model that explained 44.7% of the variance. Factors pertained to perceptions of social norms (6 items) and self-efficacy to build support and engage a team (4 items) and create (5 items) and implement (3 items) an action plan.

**Interpretation:**

We report initial development and factor analysis of a tool that the Alliance can use to assess the capacity of after-school time program providers, which is critical to targeting capacity-building interventions and assessing their effectiveness. Study findings also will inform the development of measures to assess individual capacity to plan and implement other PSE interventions.

## Background

Interventions that change organizational policies, systems, or environments (PSE interventions) are central to encouraging and supporting healthy behaviors that prevent chronic disease (1). PSE interventions include, for example, enhancing playground equipment and space, reducing access to sugar-sweetened beverages, and other interventions that “make individuals’ default decisions healthy” (1). Community-based practitioners, such as those working in departments of public health, worksites, public schools, and after-school programs, have increasing opportunities to lead and collaborate in PSE interventions (2), requiring them to develop new knowledge and skills (3). The Centers for Disease Control and Prevention, Alliance for a Healthier Generation (Alliance), and others are providing training, technical assistance, and other resources to build practitioners’ capacity, with capacity defined as practitioners’ motivation and perceptions of their ability to plan and implement PSE interventions (4,5).

Capacity building increases adoption and implementation of PSE interventions and other types of evidence-based interventions in settings where children and youths gather (6). Little is known, however, about the effect that capacity building has on individual capacity (ie, on a person’s motivation and perceptions of his or her ability) to competently plan and implement PSE interventions. This lack of knowledge is in part due to the limited number of reliable and valid measures of individual capacity (7). Measures of individual capacity are critical for programs that target training and technical assistance (TA) to reduce gaps in public health and the other practitioners’ capacity and to assess the impact that training and TA has on reducing those gaps. The purpose of our study was to collaborate with the Alliance to develop a measure of the individual capacities essential to planning and implementing PSE interventions in community out-of-school time program settings (ie, before-school, after-school, or summer learning settings). The study provides a model for an engaged approach to instrument development as well as preliminary findings on a measure of the capacity of out-of-school time program providers to plan and implement PSE interventions.

The Figure shows the conceptual framework that guided the developers of the measure of capacity of out-of-school time program providers. The framework builds on behavior change theory (5) and a review of the literature (8–11). Behavior change theory posits that people’s motivation to engage in a behavior (eg, planning and implementing a PSE intervention) is a function of their attitude toward the behavior, their beliefs about its potential outcomes, and their perceptions of how others view the behavior (ie, social norms), with particular attention to the views of those whose opinions are important to them. An individual’s ability to engage in the behavior includes having not only the confidence that they have the necessary skills and knowledge (ie, self-efficacy) but also the external resources and support needed to perform the behavior (5). The research team reviewed the literature to identify the types of knowledge and skills required to implement PSE interventions such as skill in building support among stakeholders, engaging a team, assessing the organizational context, creating and implementing an action plan, and evaluating PSE intervention outcomes (8–11).

**Figure:**
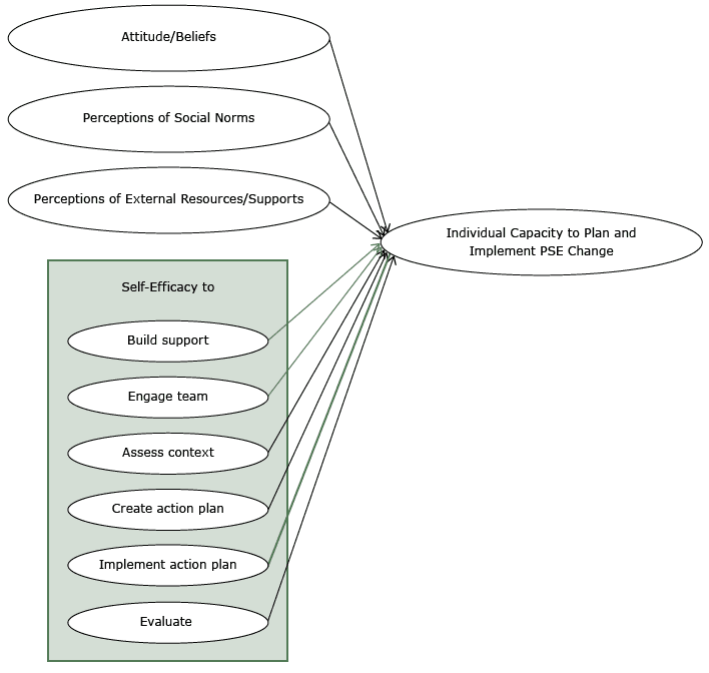
Conceptual framework used to measure the capacity of out-of-school time program providers to plan and implement interventions that change organizational policies, systems, or environments (PSE interventions).

## Community Context

The measure was developed and tested through an engaged partnership with the Alliance and staff working on the Alliance’s Healthy Out-of-School Time (HOST) Initiative. The HOST Initiative is a national program with the primary objective of fostering healthy eating and physical activity in out-of-school time programs that serve children and youths in low income communities who are at high risk of obesity, chronic disease, or food insecurity. The initiative provides out-of-school time program providers with PSE standards and best practices and with in-person coaching and online resources to help providers adapt and implement best practices to fit within the context of their out-of-school time programs. The HOST Initiative’s healthy eating and physical activity standards and best practices integrate US Department of Agriculture Smart Snacks guidelines with the National After School Association Healthy Eating and Physical Activity standards (https://www.healthiergeneration.org/_asset/pqkqhk/HOST-Framework.pdf) (12).

Through the Alliance’s HOST Initiative, 9 quality improvement coaches provide in-person support directly to providers of about 350 out-of-school time programs in 8 states. In addition, the Alliance maintains an online portal to engage other out-of-school time program sites. In total, about 2,000 sites nationwide are engaged in the HOST Initiative, most of which serve high-need children and youths (>40% of those enrolled are eligible for free or reduced-cost lunch).

We engaged with the Alliance to 1) identify and develop survey items to assess the attitudes, beliefs, skills, social influences, and resources that constitute the capacity of out-of-school time program providers to plan and implement the HOST Initiative; and 2) pilot test survey items with the goal of conducting an exploratory factor analysis to identify those items and item groupings that capture or reframe the constructs in the conceptual framework (Figure).

## Methods

### Survey development

We partnered with the Alliance to conduct formative work to create and refine an initial list of survey items. The institutional review boards at the University of North Carolina and RTI International, Inc, classified the study as exempt from review. Formative work included key informant interviews with Alliance staff, literature reviews, expert consultations, and cognitive interviews. Using the conceptual framework (Figure) as a guide, the team interviewed 2 Alliance leaders and 3 staff members to identify normative influences, resources, and skills important to planning and implementing the HOST Initiative. The team synthesized interview findings into a list of potential survey items and reviewed the literature (eg, Chinman et al [8] and Jacobs et al [13]) to find measurement instruments with relevant questions. As needed, the team revised questions and created new questions to capture themes identified in the interviews. Ten experts then reviewed the resulting list of 106 questions: 4 out-of-school time program providers with expertise implementing the HOST Initiative and 6 researchers with expertise in instrument development or in building capacity to change PSEs. After we received feedback, the list of questions was revised and reduced to 62. In the final phase of formative work, 2 individuals with expertise in cognitive interviewing conducted telephone interviews to assess each item’s interpretability and usability with providers from 10 out-of-school time programs experienced in implementing the HOST Initiative (14). Interviews were analyzed and further modifications made to the survey instrument based on results.

Respondents were asked to rate how much help they would need to perform specific behaviors (self-efficacy), how important they think participating in the HOST Initiative is to others and how important others’ opinions are to them (social norms), the adequacy of resources (resources/supports), and attitudes toward the HOST Initiative and beliefs about its potential impact (attitudes/beliefs) (Table 1). Participants responded using a 5-point Likert-type response set. Items were scored such that higher values indicated greater levels of self-efficacy, importance, resources, or agreement. The survey required all respondents to answer every question. Each item had a “Not Applicable” option, which was re-coded during the data cleaning process as missing data (15).

**Table 1:** Original Survey Items^a^ to Assess Capacity of Out-of-School Time Program Providers (n = 185) to Implement Policy, Systems, and Environmental Change, Alliance for a Healthier Generation’s Healthy Out-of-School Time (HOST) Initiative

Question/Item	Description	Mean	Median	Skewness Statistic^b^	Communality Score^c^
**How much help would you need to do the following? (self-efficacy)**					
1	Talk to parents about the benefits of HOST	3.76	4	−0.49	0.32
2^d^	Talk to coworkers about the benefits of HOST	4.37	5	−1.61	0.58
3^d^	Persuade my site’s leadership of the benefits of HOST	3.98	4	−0.63	0.47
4	Describe HOST process, standards, and practices	3.51	3	−0.03	0.26
5	Work with community partners to strengthen my after-school site’s work with HOST	3.68	4	−0.27	0.28
6^d^	Involve team members in making decisions about planning and implementing HOST	3.97	4	−0.54	0.51
7^d^	Delegate tasks for HOST to coworkers	4.12	4	−0.9	0.49
**Please rate how important you think participating in the HOST Initiative is to others. (social norms)**					
8	My coworkers (at my site)	3.55	4	−0.24	0.28
9	My supervisor	3.93	4	−0.53	0.39
10	My program leadership	3.88	4	−0.16	0.39
11	Other after-school programs in my community	3.34	3	−0.09	0.28
12	My program’s funders	4.04	4	−0.51	0.49
13	My national organization	4.50	5	−1.18	0.34
**For each person or group listed, please rate how important their opinion about the HOST Initiative is to you. (social norms)**					
14^d^	To my coworkers (at my site)	4.20	4	−1.06	0.60
15^d^	To my supervisor	4.43	5	−1.15	0.89
16^d^	To my program leadership	4.46	5	−1.24	0.93
17^d^	To other after-school programs in my community	3.84	4	−0.61	0.78
18^d^	To my program’s funders	4.54	5	−1.36	0.88
19^d^	To my national organization	4.63	5	−1.42	0.62
**How much help would you need to do the following? (self-efficacy)**					
20	Use HOST online inventory to describe healthy eating and physical activity practices at my site	3.86	4	−0.38	0.30
21	Use data from the HOST online inventory to identify gaps in my site’s healthy eating and physical activity practices	3.69	4	−0.18	0.41
22	Work with my team members to select action items for our HOST action plan	4.08	4	−0.36	0.42
23	Work with my team members to develop goals and objectives for achieving our action plan	3.95	4	−0.6	0.35
24^d^	Create a timeline for completing the steps in my site’s HOST action plan	3.91	4	−0.29	0.54
25^d^	Describe the resources required to complete the steps in my site’s HOST action plan	3.74	4	−0.27	0.49
26^d^	Access resources on the Alliance for a Healthier Generation website	4.08	4	−0.49	0.75
27^d^	Access local resources to support my site’s work with HOST	3.79	4	−0.33	0.59
28	Obtain funding to support my site’s work with HOST	3.06	3	−0.03	0.40
29^d^	Get help from experts at the Alliance for a Healthier Generation for training and support	3.62	4	−0.2	0.81
30^d^	Stick to a timeline for completing the steps in a HOST action plan	3.87	4	−0.34	0.78
31^d^	Hold members of my site team accountable for completing their assigned HOST tasks	3.97	4	−0.39	0.81
32^d^	Work with team members at my site to solve problems that occur while implementing our HOST action plan	4.07	4	−0.48	0.76
33	Assess my site’s progress toward attaining the goals and objectives identified in the action plan	4.06	4	−0.58	0.59
34	Write reports about my after-school site’s work with HOST	3.93	4	−0.59	0. 30
35	Communicate the success of my after-school site’s work with HOST	4.06	4	−0.68	0.26
36	Use my after-school site’s success with HOST to increase support for the program	3.97	4	−0.43	0.33
**Please rate the adequacy of the following resources. (resources/supports)**					
37	Space for HOST nutrition and fitness activities	3.91	4	−0.89	0.10
38	Equipment for HOST nutrition and fitness activities	3.67	4	−0.44	0.07
39	Time for planning for HOST	3.37	4	−0.34	0.33
40	Funds available for HOST	2.68	3	0.18	0.29
41	Support from site leadership for HOST	3.77	4	−0.49	0.18
42	Team members available to work on HOST	3.33	3	−0.19	0.32
43	How much authority (power) do I feel I have to coordinate HOST?	3.82	4	−0.73	0.19
**How much do you agree or disagree with the next 2 statements? (attitude/beliefs)**					
44	My after-school site’s work with HOST will take attention away from other high-priority activities	2.48	2	0.41	0.08
45	Staff at my after-school site actively seek new ways to improve how we do things	3.88	4	−0.44	0.30
**What impact do you believe the HOST standards and best practices will have on . . . (attitude/beliefs)**					
46	Presence of healthy foods and beverages in my after-school site?	3.5	4	−0.26	0.24
47	Opportunities for children to be physically active in my after-school site?	3.64	4	−0.23	0.27

a Participants responded using a 5-point Likert-type response set. Items were scored such that higher values indicated greater levels of self-efficacy, importance, resources, or agreement. The survey required all respondents to answer every question. Each item had a “Not Applicable” option, which was re-coded during the data cleaning process as missing data (15).

b Calculated using the Fisher-Pearson standardized moment coefficient, part of the descriptive statistics included in the R software package (R version 3.2 and R Studio version 0.98.1103 [R Foundation for Statistical Computing]).

c Communality scores indicate the portion of a variable’s variance that is common to the extracted factor. Items with a score <0.30 were dropped to improve the suitability of the item set for further analysis.

d Item that contributed to 1 of the 4 final factors.

### Survey administration

Potential participants were 345 out-of-school time program providers employed by National Recreation and Park Association or the Boys and Girls Clubs of America affiliated programs that were responsible for coordinating implementation of the Alliance’s HOST Initiative. One individual was recruited to participate from each out-of-school time program site. Survey administration took place between December 2014 and February 2015. An invitation e-mail and 3 bi-weekly email reminders were sent to potential participants with a direct link to the online survey. Participants received a $10 gift card to a major online retailer upon survey completion. The final sample was 185 individuals (53% response rate) with 145 (78%) completing the survey. After deleting surveys with missing data (ie, use of “Not Applicable” response), the analysis data set included 104 complete observations.

### Data analysis

We examined whether missing information (ie, incomplete responses) could contribute to a biased interpretation of the data. Little’s Missing Completely at Random (MCAR) test indicated that there was no discernable pattern of missing data (χ^2^_1400df_ = 1464.32, *P* < 0.11), which supports the use of listwise deletion to remove incomplete cases. Using listwise deletion resulted in an analysis sample of data from 104 respondents who provided information on all variables that assess capacity to plan and implement the HOST Initiative.

Next, we conducted an exploratory factor analysis in 4 steps. We used principal factor analysis (PFA) with promax rotation. In the first step, we evaluated the suitability of the 47-item correlation matrix for factor extraction by using the Kaiser-Meyer-Olkin (KMO) measure of sampling adequacy. KMO values below 0.60 are poor, values of 0.60 or greater but less than 0.80 are acceptable, and values at 0.80 or greater are meritorious (16). The initial PFA yielded a KMO score of 0.49, indicating that the total item set was poorly suited to further analysis. In the second step, we removed 16 items with KMO values of 0.30 or lower and reran the PFA. This yielded a KMO value of 0.79, well above the acceptable range and approaching a rating of meritorious.

In the third step, we used parallel analysis (17) to determine the number of factors within the data. Parallel analysis is preferable to Kaiser’s eigenvalue-greater-than-1 rule for determining the appropriate number of factors to extract when conducting exploratory factor analysis (18). The parallel analysis suggested a 4-factor solution. The initial eigenvalues for the 4 factors ranged from 11.89 to 1.29, and the 4-factor solution explained 44.7% of the total variance before rotation. Items were considered part of a factor if item factor loading was equal to or greater than 0.60 (19). None of the items had to be removed due to cross loading on more than 1 factor. Eighteen of the 31 items contributed to the 4 factors identified; the remaining 13 items did not have item loadings above the 0.60 threshold for any factor. These items are not discussed further. In the fourth step, we examined ordinal α (20) scores to determine the factor structure (ie, to identify the items that loaded within each factor). Data were analyzed by using Stata 13.1 (StataCorp LP) and R (version 3.2) and R Studio (version 0.98.1103) (R Foundation for Statistical Computing).

## Outcomes

For the first factor, social norms, we used 6 items to ask respondents to rate how important other people’s (eg, coworkers, supervisor) opinion of HOST was to them (Table 2). The second factor, create an action plan, had 5 items related to self-efficacy to perform behaviors that occur before implementation (eg, create a timeline, access local resources). The third factor, implement an action plan, had 3 items related to self-efficacy to perform behaviors that occur during implementation (eg, stick to a timeline, hold team members accountable). The fourth factor, engage stakeholders, combines 4 items from 2 of the self-efficacy constructs: build support for working on the HOST Initiative and engage the team.

**Table 2:** Final Factors for a Tool to Assess Capacity of Out-of-School Time Program Providers(n = 104) to Implement Policy, Systems, or Environment Change, Alliance for a Healthier Generation’s Healthy Out-of-School Time (HOST) Initiative

Factor	Question	Scale Alpha	Item Alpha^a^	Respondent Rating^b^
Social norms	**How important others opinions of HOST Initiative are to you**			
	Coworkers	0.91	0.90	79.5%
	Supervisor		0.89	87.9%
	Program leadership		0.89	87.9%
	Other after-school programs		0.90	66.7%
	Program funders		0.88	86.4%
	National organization		0.90	93.2%
Create action plan	**How much help would you need to . . .**			
	Create a timeline	0.90	0.86	70.2%
	Describe resources required		0.86	63.8%
	Access resources on Alliance web site		0.87	72.3%
	Access local resources		0.87	61.7%
	Get help from experts at Alliance		0.90	56.7%
Implement action plan	**How much help would you need to . . .**			
	Stick to a timeline	0.90	0.88	68.3%
	Hold team members accountable		0.86	69.0%
	Work with team to solve problems		0.85	76.6%
Engage stakeholders	**How much help would you need to . . .**			
	Talk to coworkers about benefits	0.86	0.85	87.9%
	Persuade leadership of benefits		0.81	72.1%
	Involve team members in decisions		0.80	72.1%
	Delegate tasks		0.81	80.7%

a Reported estimates of intercorrelation are based on the ordinal version of coefficient α.

b Respondents’ rating of social norms was the percentage who viewed each item as a high or very high priority (ie, gave the item a rating of 4 or 5 on a 5-point Likert-type response set). For the other factors, the rating is the percentage who reported they could do the task with little or no help (ie, gave the item a rating of 4 or 5 on a 5-point Likert-type response set).

Two constructs of the study’s conceptual framework (attitudes/beliefs and assess context) (Figure), were not supported as scales because each had only 2 items in the final analysis. The analysis also did not support 2 other constructs (evaluate and perceptions of external resources/supports).

## Interpretation

The analysis identified 4 capacity-related constructs and 18 survey items for use in assessing those constructs. The 4 constructs incorporate 5 of the 9 constructs in the original conceptual framework (2 constructs were combined). One factor pertained to the perceptions out-of-school time program providers with regard to social norms, and 3 factors pertained to providers’ self-efficacy to perform specific behaviors (create an action plan, implement an action plan, and engage stakeholders).

The study was limited by its small sample and by its focus on individuals already engaged in implementing the HOST Initiative. Additional research is needed to confirm the identified factors among both those who are and those who are not yet working to implement the HOST Initiative. Research also is needed to further test the factors that were not supported in this analysis. Attitudes/beliefs is a central construct in behavior change theory (5), and assessing contexts and evaluation repeatedly are important behaviors in PSE change (9). These constructs may not have been supported because the study had an inadequate sample size or because the constructs are less important to implementing the HOST Initiative than they are to other PSE interventions.

PSE interventions are essential to supporting healthier behaviors; yet the science of how to build community practitioners’ capacity to implement those behaviors is just emerging (6). Governments and foundations are investing funds in PSE interventions, resulting in a pressing need for measures of practitioners’ capacity to do this work.

Instruments exist to assess practitioners’ capacity to plan and implement evidence-based interventions (8,9). These instruments ask broad questions such as how strongly participants agree that they “have the skills necessary for developing evidence-based chronic disease control programs” (9). Although broad questions have the strength to identify baseline competencies, they may not get at the specific skills, beliefs, resources, and social influences affecting practitioners’ capacity to implement PSE interventions within distinct contexts, such as out-of-school time program settings. Our study describes an approach that can be used to engage both the providers and recipients of capacity-building interventions in identifying the skills and resources that community practitioners require and the beliefs and social influences that motivate them to plan and implement PSE interventions.

The survey developed in this study is the first step in creating an instrument that might be used to assess gaps in the capacity of providers of out-of-school time programs participating in the HOST Initiative, information that might be used to target capacity building to address areas of greatest need. For example, efforts to develop capacity might target the factors (eg, create an action plan) about which the fewest practitioners reported feeling confident. An instrument such as the one we are developing also might be used to assess the effects that capacity building have on practitioners’ capacity, information that is essential to tailoring capacity building and assessing the results over time. Additional confirmatory factor analysis of the questionnaire with providers of out-of-school time programs who implement the HOST Initiative will inform further development of this measure and adaptation. Research also is needed to assess whether the instrument’s measures are associated with improvement in adoption and implementation of PSE interventions within out-of-school time program settings.
